# Intravenous Phosphate Loading Increases Fibroblast Growth Factor 23 in Uremic Rats

**DOI:** 10.1371/journal.pone.0091096

**Published:** 2014-03-13

**Authors:** Noriko Arai-Nunota, Masahide Mizobuchi, Hiroaki Ogata, Ai Yamazaki-Nakazawa, Chiaki Kumata, Fumiko Kondo, Nozomu Hosaka, Fumihiko Koiwa, Eriko Kinugasa, Takanori Shibata, Tadao Akizawa

**Affiliations:** 1 Division of Nephrology, Department of Medicine, Showa University School of Medicine, Tokyo, Japan; 2 Department of Internal Medicine, Showa University Northern Yokohama Hospital, Yokohama, Japan; 3 Division of Nephrology, Department of Medicine, Showa University Fujigaoka Hospital, Yokohama, Japan; University of Navarra School of Medicine and Center for Applied Medical Research (CIMA), Spain

## Abstract

Oral phosphate loading and calcitriol stimulate Fibroblast growth factor 23 (FGF23) secretion, but the mechanisms underlying the stimulation of FGF23 remain to be studied. We compared the effect of intravenous phosphate loading with that of oral loading on FGF23 levels in normal and 5/6 nephrectomized uremic rats. Uremic rats (Nx) and sham-operated rats were fed a normal phosphate diet for 2 weeks and then divided into 3 groups: 1) with the same phosphate diet (NP), 2) with a high phosphate diet (HP), and 3) NP rats with intravenous phosphate infusion using a microinfusion pump (IV). Blood and urine were obtained 1 day (early phase) and 7 days (late phase) after the interventions. In the early and late phases, serum phosphate levels and fractional excretion of phosphate (FEP) were comparable in the HP and IV groups in both Sham and Nx rats. Serum phosphate levels in the HP and IV groups were equally and significantly higher than those in the NP group only in the late phase in Nx rats. In the early phase, FGF23 levels were comparable in the NP, HP, and IV groups, but were significantly higher in the HP and IV groups compared to the NP group in the late phase in Nx rats. 1α-hydroxylase and sodium dependent phosphate co-transporter 2a expression levels in the kidney in Nx rats were equally and significantly decreased in the HP and IV groups compared with the NP group, while 24-hydroxylase expression was equally and significantly increased. These results show that chronic intravenous phosphate loading increases bioactive FGF23, indicating that an alternative pathway for FGF23 regulation, in addition to the dietary route, may be present. This pathway is clearer under conditions produced by a kidney injury in which phosphate is easily overloaded.

## Introduction

Phosphate burden resulting in hyperphosphatemia is associated with mortality in patients with chronic kidney disease (CKD), regardless of dialysis treatment [1], [2]. Thus, maintenance of phosphate homeostasis is important in these patients. The kidney is the major organ that regulates phosphate homeostasis through hormones such as parathyroid hormone (PTH), 1,25-dihydroxyvitamin D [1,25(OH)_2_D], and the more recently identified fibroblast growth factor-23 (FGF23).

FGF23 is produced mainly by osteocytes and prevents phosphate overload by inducing phosphaturia through inhibition of sodium dependent phosphate co-transporters (NPT) 2a and 2c, and mediation of vitamin D metabolism, in which FGF23 induces inhibition of 1α-hydroxylase and stimulation of 24-hydroxylase [3]. Evidence is growing that FGF23 plays a crucial role in phosphate metabolism in patients with CKD. FGF23 elevation in response to loss of renal function is the initial step leading to 1,25(OH)_2_D reduction and PTH elevation in CKD [4], [5]. The decline in 1,25(OH)_2_D increases PTH production, which acts additively with FGF23 to stimulate phosphaturia and prevent phosphate overload. In addition to the association with phosphate metabolism, FGF23 has been linked to progression of renal disease [6] and cardiovascular dysfunction [7]–[9] in CKD patients, with or without dialysis. Elevated FGF23 is also associated with increased mortality, independent of serum phosphate levels [10]. Recently, a toxic effect of FGF23 contributing to left ventricular hypertrophy has also been shown [11]. These findings emphasize the importance of physiologic research on FGF23.

While the association of FGF23 with various disease conditions, including those beyond phosphate metabolism, has been demonstrated, little is known about the regulation of FGF23. Proposed regulators of FGF23 include oral phosphate loading [12]–[14], 1,25(OH)_2_D [15], PTH [16], [17], and metabolic acidosis [18], which increase FGF23 in normal kidney function. However conflicting results showing that these factors do not affect FGF23 have also been reported [19]–[21], and 1,25(OH)_2_D and dietary phosphate increase FGF23 in a uremic condition [22]. Based on these findings, further investigation is needed to clarify the mechanism by which FGF23 is primarily regulated. To this end, we studied the effect of intravenous (IV) phosphate loading on FGF23 in normal and 5/6 nephrectomized uremic rats. Our results showed that continuous IV phosphate loading, as well as oral phosphate loading, increased bioactive FGF23 in uremic rats.

## Materials and Methods

### Experimental Protocol

All studies were approved by the Showa University Animal Studies Committee in accordance with federal regulations. Renal insufficiency was induced by 5/6 nephrectomy (Nx) in a group of male Sprague-Dawley rats weighing 225–250 g. Nx involves ligation of several branches of the left renal artery and excision of the right kidney. All rats were fed a normal phosphate (NP, phosphate 0.5%, calcium 0.8%, and vitamin D1000IU/kg) diet. Experiments were started 2 weeks after the Nx or sham operation (Sham) to allow time for recovery. At this time, an infusion mini pump (iPRECIO, Primtech Corp., Tokyo, Japan) was implanted into some Nx and Sham rats, after which saline was infused for 7 days and phosphate solution was infused for another 7 days through a catheter placed in the right jugular vein. The phosphate solution contained NaH_2_PO_4_ and Na_2_HPO_4_ (1∶2) to achieve a final phosphate concentration of 2 M. Rats that underwent IV phosphate infusion were fed the NP diet throughout the study period (Nx+IV and Sham+IV rats). The 2 M phosphate solution was injected continuously at 20 µL/hr to give a daily amount of phosphate of about 91 mg, corresponding to 230 mg/kg body weight, and a fluid volume of 480 µL. Other Nx and Sham rats were fed a high phosphate (HP, phosphate 0.8%, Calcium 0.8%, and vitamin D 1000 IU/kg) diet for 7 days (Nx+HP and Sham+HP rats) with the same timing as for intravenous phosphate infusion in the IV groups. Control Nx and Sham rats were fed the NP diet throughout the study (Nx+NP and Sham+NP rats). For the first and last day of the phosphate loading period, rats were placed in metabolic cages and 24-h urine samples were collected to permit calculation of 24-h creatinine clearance (24 h Ccr) and fractional excretion of phosphate (FEP). FEP was calculated using the formula: [urine phosphate×serum creatinine]×100/[serum phosphate×urine creatinine]. One day after the start of phosphate loading, 1 ml of blood was collected from the left jugular vein (the opposite side to catheterization) to determine changes in parameters in the early phase of the study. After 7 days of phosphate loading, all rats were sacrificed by exsanguination via the dorsal aorta. The kidney was removed and cut into coronal pieces for histological examination.

### Analytical Determinants

Urine and serum levels of albumin, creatinine, calcium, and phosphate were measured with commercially available kits. Serum PTH and FGF23 levels were determined using a Rat Bioactive Intact PTH ELISA Kit (Immutopics, Inc. San Clemente, CA, USA) and an Intact FGF23 Assay Kit (Kainos, Tokyo, Japan), respectively.

### Immunohistochemistry

The kidney was fixed in 10% formalin overnight, embedded in paraffin, and cut into 4-µm coronal sections for assessment of expression of 1α-hydroxylase, 24-hydroxylase, and NPT-2a. The sections were deparaffinized, rehydrated, and microwaved in 0.01 mol/L citrate buffer (pH 6.0) for 10 min to retrieve antigens. The sections were then treated with 0.6% hydrogen peroxide in methanol for 10 min at room temperature to block endogenous peroxidase and subsequently blocked with 10% pre-immune goat serum for 30 min at room temperature. A primary rabbit anti-1α-hydroxylase antibody (Santa Cruz Biotechnology, Inc. Santa Cruz, CA, USA, 1∶200 dilution), rabbit anti-24-hydroxylase antibody (Santa Cruz Biotechnology, 1∶1000 dilution), rabbit anti-NPT-2a antibody (Alpha Diagnostic International, San Antonio, TX, USA, 1∶50 dilution) or pre-immune IgG was added, followed by incubation at room temperature for 2 h. The biotinylated secondary antibody was applied, followed by a streptavidin-HRP conjugate. Immune complexes were visualized with 3-amino 9-ethylcarbazole substrate-chromagen. Finally, all sections were counterstained with hematoxylin.

### Western Blot Analysis

Protein expression levels of 1α-hydroxylase, 24-hydroxylase, and NPT-2a were quantified by western blot analysis. Briefly, renal cortex tissue was homogenized in 2 ml of Cell Lysis Buffer (Cell Signaling Technology, Beverly, MA). Samples were centrifuged at 3000×*g* for 15 min and the supernatants were assayed. After being mixed with SDS-PAGE sample buffer and boiled for 5 min, samples (5 µg per lane) were electrophoresed on 4 to 12% SDS polyacrylamide gels and transferred to nitrocellulose membranes for 2 h at 30 V. Membranes were blocked for 30 min with Tris-buffered saline containing 5% BSA (5% BSA/TBS) and incubated with diluted primary antibody overnight at room temperature in 5% BSA/TBS containing 0.05% Tween 20. The source and concentration of each antibody were as follows: rabbit anti-1α-hydroxylase (Santa Cruz Biotechnology; 1∶200), rabbit anti-24-hydroxylase, goat anti-NPT2a (Santa Cruz Biotechnology; 1∶100), and rabbit anti- glyceraldehyde-3-phosphate dehydrogenase (GAPDH) (Cell Signaling Technology; 1∶500). The membranes were washed and diluted secondary horseradish peroxidase (HRP)-conjugated antibodies were added (anti-rabbit IgG-HRP 1∶1000; anti-goat IgG-HRP 1∶1000; Santa Cruz Biotechnology). The membranes were washed again and developed using an enhanced chemiluminescence system (ECL Prime Western Blotting Detection Reagent; GE Healthcare UK Ltd., Amersham Place, Little Chalfont, Buckinghamshire, England). Changes in 1α-hydroxylase, 24-hydroxylase, and NPT-2a expression were normalized by correction for the densitometric intensity of GAPDH for each sample.

### Statistical Analysis

Data for each experimental group are expressed as mean ± s.e. Analysis of variance was used and *post hoc* pairwise comparisons of group means were performed using a Tukey-Kramer honestly significant difference test. Differences between groups were considered significant at a *P* value of <0.05.

## Results

### Effects of Intravenous Phosphate Loading on Serum Chemistry

Serum chemistry data for sham rats with oral or IV phosphate loading are shown in Table 1. Serum creatinine levels were similar in all three groups (Sham+NP, Sham+HP, and Sham+IV rats) throughout the study period, indicating that phosphate loading had a minor effect on kidney function. Serum phosphate and PTH levels on days 1 and 7 equally and marginally increased in Sham+HP and Sham+IV rats, compared with the respective values in Sham+NP rats. Compared with Sham+NP rats, FGF23 levels in Sham+HP and Sham+IV rats were significantly increased on day 1. There were no significant differences among the three groups on day 7.

**Table 1:** Serum chemistries of sham rats (Day 1 and Day 7).

	BW	Cre	P	PTH	FGF23
Day 1	g	mg/dl	mg/dl	pg/ml	pg/ml
Sham+NP (n = 9)	413±14	0.1±0.0	8.2±0.2	32±7	375±45
Sham+HP (n = 9)	419±12	0.2±0.0	8.4±0.3	50±7	674±83#
Sham+IV (n = 10)	401±15	0.2±0.0	8.5±0.2	54±8	625±69#
**Day 7**					
Sham+NP (n = 9)	426±14	0.2±0.0	7.9±0.3	37±5	313±44
Sham+HP (n = 9)	428±11	0.2±0.0	8.2±0.3	62±8	492±73
Sham+IV (n = 10)	410±12	0.2±0.0	8.4±0.3	62±14	494±44

Data are means±s.e.; Cre, serum creatinine; P, serum phosphate; PTH, parathyroid hormone; FGF, fibroblast growth factor; NP, normal phosphate diet; HP, high phosphate diet; IV, intravenous phosphate load.

# P<0.05 versus Sham+NP (Day 1).

Serum chemistry data for Nx rats with oral or IV phosphate loading are shown in Table 2. Similarly to Sham rats, serum creatinine levels were similar in all three groups (Nx+NP, Nx+HP, and Nx+IV rats). Serum phosphate levels were significantly increased only on day 7 in Nx+HP and Nx+IV rats compared with those in Nx+NP rats. The levels were similar in Nx+HP and Nx+IV rats. FGF23 was significantly increased in Nx+HP compared with that in Nx+NP rats on day 1, but was equally and significantly increased in Nx+HP and Nx+IV rats compared with Nx+NP rats on day 7. PTH in Nx rats showed a similar trend to that in Sham rats.

**Table 2:** Serum chemistries of uremic rats (Day 1 and Day 7).

	BW	Cre	P	PTH	FGF23
Day 1	G	mg/dl	mg/dl	pg/ml	pg/ml
Nx+NP (n = 6)	377±11	0.5±0.0	8.7±0.3	56±9	613±33
Nx+HP (n = 7)	376±12	0.5±0.0	9.1±0.2	83±11	846±80†
Nx+IV (n = 8)	380±4	0.6±0.0	9.0±0.3	121±31	773±45
**Day 7**					
Nx+NP (n = 6)	396±14	0.5±0.0	8.2±0.2	107±36	438±39
Nx+HP (n = 7)	419±14	0.5±0.0	9.3±0.3$	173±53	831±64$
Nx+IV (n = 8)	393±10	0.6±0.0	9.6±0.2$	141±16	1070±110$

Data are means ±s.e.; Cre, serum creatinine; P, serum phosphate; PTH, parathyroid hormone; FGF, fibroblast growth factor; Nx, 5/6 nephrectomized; NP, normal phosphate diet; HP, high phosphate diet; IV, intravenous phosphate load.

† P<0.05 versus Nx+NP (Day 1),

$ P<0.05 versus Nx+NP (Day 7).

### Effects of Intravenous Phosphate Loading on Kidney Function

Urine data for Sham and Nx rats are shown in Tables 3 and 4, respectively. The 24 hr Ccr level was similar in all three groups on days 1 and 7 in Nx rats and Sham rats. Nx+HP and Nx+IV rats showed equal and significant increases in FEP compared with Nx+NP rats on days 1 and 7. A similar trend was observed in Sham rats.

**Table 3:** Urinary data of sham rats (Day 1 and Day 7).

	UV	24 hrCcr	FEP
Day 1	ml/day	ml/min	%
Sham+NP (n = 9)	11±2	5.9±0.6	2±0
Sham+HP (n = 9)	12±2	5.0±0.3	8±1#
Sham+IV (n = 10)	23±2#	5.4±0.5	7±1#
**Day 7**			
Sham+NP (n = 9)	16±2	6.2±0.6	2±0
Sham+HP (n = 9)	16±3	5.3±0.5	9±1 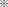
Sham+IV (n = 10)	19±4	5.8±0.5	9±1 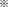

Data are means±s.e.; UV, urine volume; Ccr, creatinine clearance; FEP, fractional excretion of phosphate; NP, normal phosphate diet; HP, high phosphate diet; IV, intravenous phosphate load.

# P<0.05 versus Sham+NP (Day 1),

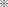
P<0.05 versus Sham+NP (Day 7).

**Table 4:** Urinary data of uremic rats (Day 1 and Day 7).

	UV	24 hrCcr	FEP
Day 1	ml/day	ml/min	%
Nx+NP (n = 6)	10±1	1.7±0.3	8±1
Nx+HP (n = 7)	14±4	1.4±0.1	23±2†
Nx+IV (n = 8)	18±2	1.5±0.1	24±2†
**Day 7**			
Nx+NP (n = 6)	13±1	1.9±0.3	9±2
Nx+HP (n = 7)	17±3	1.7±0.1	30±3$
Nx+IV (n = 8)	16±2	1.4±0.1	32±4$

Data are means ±s.e.; UV, urine volume; Ccr, creatinine clearance; FEP, fractional excretion of phosphate; NP, normal phosphate diet; HP, high phosphate diet; IV, intravenous phosphate load.

† P<0.05 versus Nx+NP (Day 1),

$ P<0.05 versus Nx+NP (Day 7).

### Bioactivities of FGF23 Induced by Intravenous Phosphate Loading

**Figure 1:**
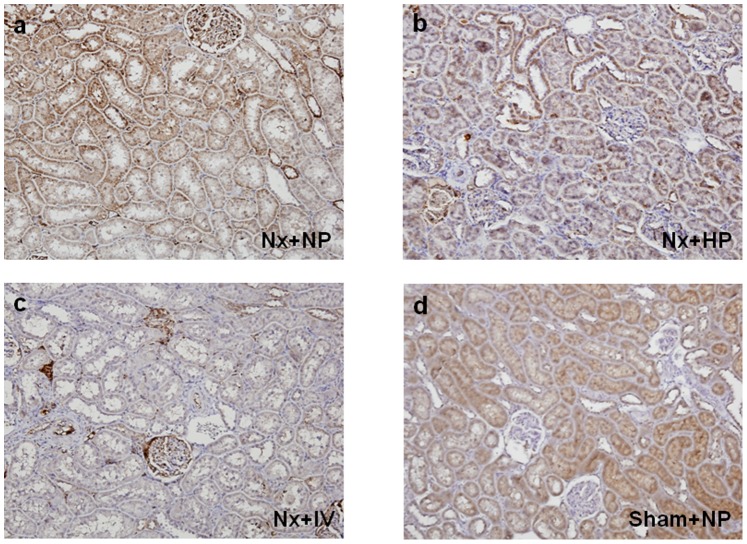
Effect of phosphate loading on 1α-hydroxylase expression in the kidney of uremic rats on day 7. (a) Nx+NP, (b) Nx+HP, (c) Nx+IV rats, and (d) Sham+NP rats. Magnification×100.

**Figure 2:**
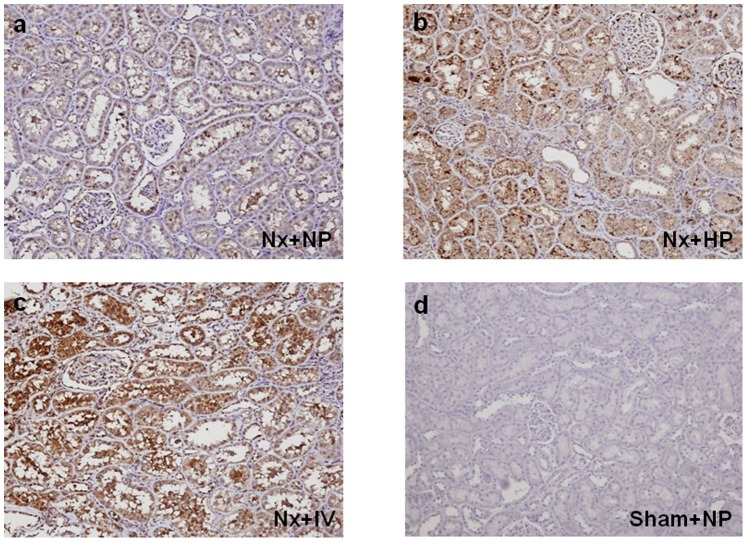
Effect of phosphate loading on 24-hydroxylase expression in the kidney of uremic rats on day 7. (a) Nx+NP, (b) Nx+HP, (c) Nx+IV rats, and (d) Sham+NP rats. Magnification×100.

**Figure 3:**
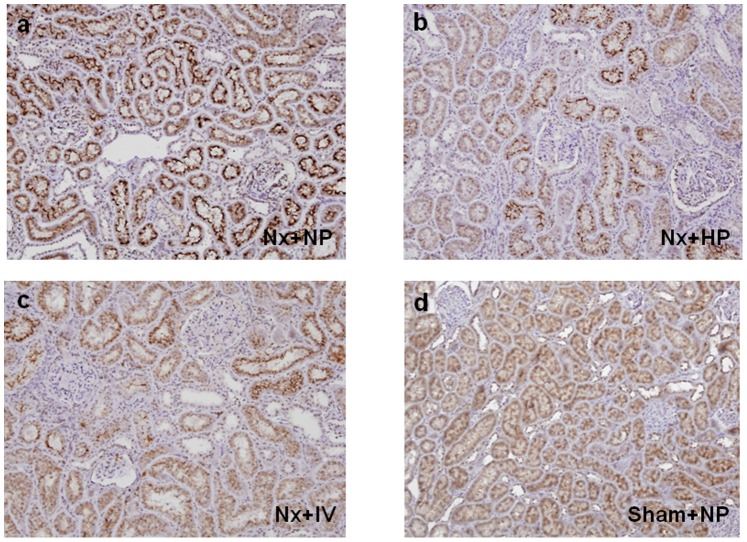
Effect of phosphate loading on NPT-2a expression in the kidney of uremic rats on day 7. (a) Nx+NP, (b) Nx+HP, (c) Nx+IV rats, and (d) Sham+NP rats. Magnification×100.

**Figure 4:**
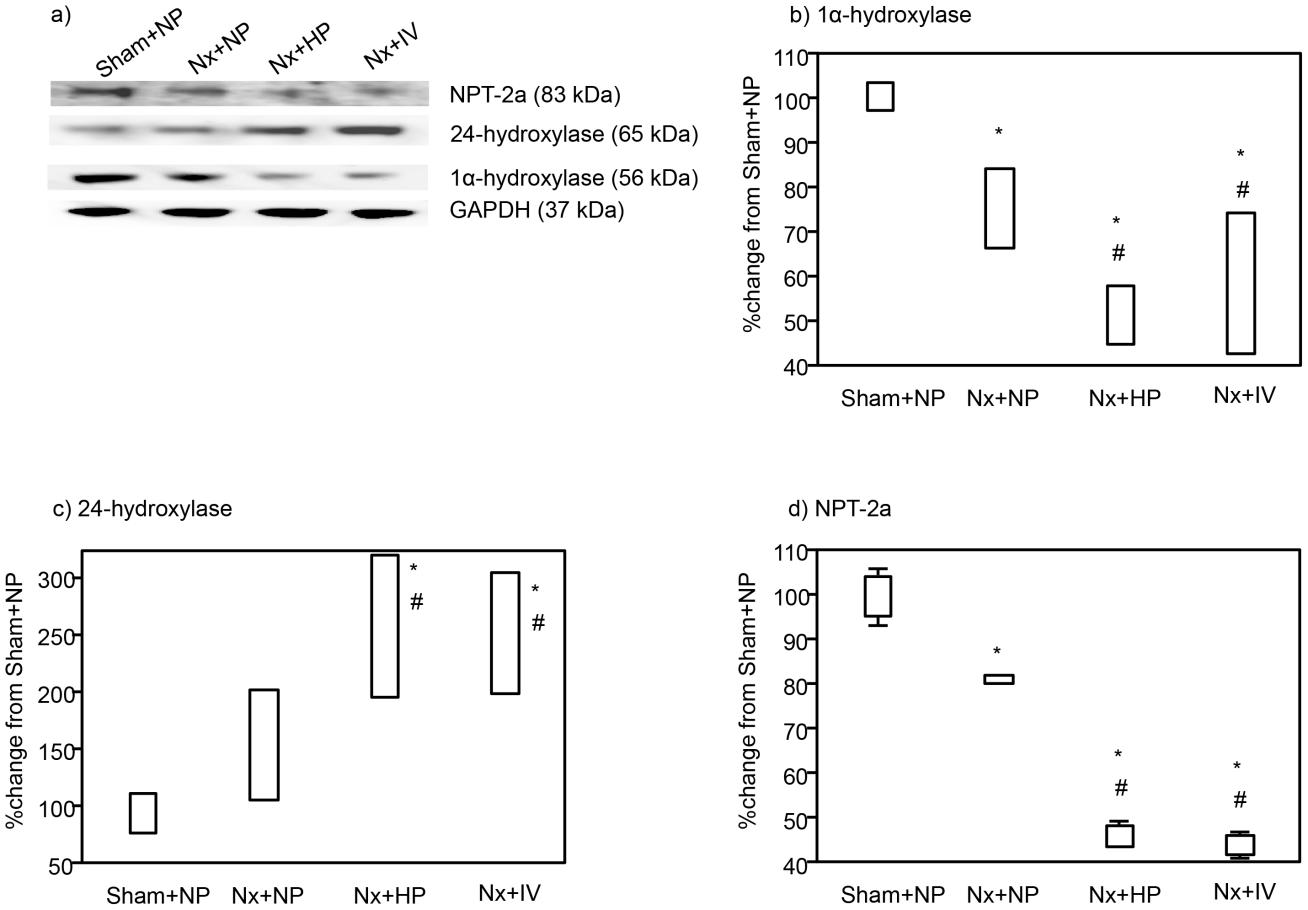
Immunoblotting for 1α-hydroxylase, 24-hydroxylase, NPT-2a, and glyceraldehyde-3-phosphate dehydrogenase (GAPDH) (a). Densitometric quantification of the corresponding bands was performed using an image analyzer, 1α-hydroxylase (b) 24-hydroxylase (c), and NPT-2a (d). Data are presented after normalization to GAPDH expression and are depicted as the percentage change from the respective controls (Sham+NP). Data are shown as mean ± s.e. (*n* = 4 each). *P<0.05 vs. Sham+NP and ^#^P<0.05 versus Nx+NP.

The effects of continuous (day 7) oral and IV phosphate loading on expression of 1α-hydroxylase (Figure 1), 24-hydroxylase (Figure 2), and NPT-2a (Figure 3) were examined in kidney tissues in Nx rats. Oral and IV phosphate loading induced decreases in 1α-hydroxylase (Figure 1b and 1c) and NPT-2a (Figure 3b and 3c), and increased 24-hydroxylase expression (Figure 2b and 2c), in association with FGF23 elevation. Western blotting showed similar results to those of immunohistochemistry. 1α-hydroxylase (Figure 4b) and NPT-2a (Figure 4d) in Nx+NP rats were significantly lower than those in Sham+NP rats, while 24-hydroxylase (Figure 4c) in Nx+NP rats showed a moderate increase compared with Sham+NP rats. 1α-hydroxylase and NPT-2a in Nx+HP and Nx+IV rats were equally and significantly decreased (Nx+HP: 1α-hydroxylase 52% of Sham+NP, P<0.05, NPT-2a 46% of Sham+NP, P<0.05; Nx+IV: 1α-hydroxylase 54% of Sham+NP, P<0.05, NPT-2a 45% of Sham+NP, P<0.05) compared with Nx+NP rats (1α-hydroxylase 76% of Sham+NP, NPT-2a 80% of Sham+NP). In contrast, 24-hydroxylase was significantly increased in Nx+HP (256% of Sham+NP, P<0.05) and Nx+IV (269% of Sham+NP, P<0.05) rats compared with Nx+NP rats (155% of Sham+NP).

## Discussion

Disturbance of phosphate metabolism is strongly associated with mortality in patients with CKD and careful attention to phosphate metabolism is required in these patients. Along with 1,25(OH)_2_D and PTH, FGF23 has become a central player in this field. FGF23 has been associated with various morbidities and mortality in CKD, but the precise mechanisms through which FGF23 is regulated remain unclear. Our results demonstrate that continuous IV phosphate loading increased serum FGF23 to the same extent as oral phosphate loading in 5/6 nephrectomized uremic rats. Furthermore, the increased FGF23 was bioactive, as shown by its effects on 1α-hydroxylase, 24-hydroxylase, and NPT-2a expression levels in kidney tissues.

The main factors that increase FGF23 are oral phosphate loading, 1,25(OH)_2_D, and PTH [3]. In normal kidney function, oral phosphate loading has various effects on FGF23. Dietary phosphate intake has been found to increase FGF23 in humans [13], [14], [23] and mice [12], but has also been shown not to increase FGF23 in humans [19], [20]. The apparently variable effects of oral phosphate loading on FGF23 may have several explanations. First, an increase in FGF23 by high phosphate diets is probably mediated by the 1,25(OH)_2_D-vitamin D receptor (VDR) pathway because dietary phosphate does not increase FGF23 in the absence of VDR [24]. Second, the observation period may have been too short to detect changes in FGF23 in studies that failed to find an increase in FGF23.

A high phosphate diet increases FGF23 in patients with renal impairment [22] and manipulation of oral phosphate intake using a phosphate binder regulates FGF23 in uremic rats [25], suggesting that renal impairment is favorable for oral phosphate loading to increase FGF23. In the present study, both IV and oral phosphate loading showed a tendency to increase FGF23 in sham rats, but caused a significant increase in FGF23 in Nx rats. However, elevation of FGF23 in the uremic rats with IV or oral phosphate loading was only clear in the late phase (day 7), and not in the early phase (day 1). In sham rats with oral or IV loading, FGF23 on day 7 was slightly (but not significantly) decreased compared with that on day 1. This may suggest that phosphate overload is properly compensated for by phosphaturic factors other than FGF23, leading to minor requirement for FGF23 elevation in normal kidney function.

The main purpose of the study was to investigate the effects of intravenous phosphate loading on FGF23 in rats with normal and impaired kidney function. However, it is also of interest to consider the effect of kidney injury on FGF23 under each phosphate loading condition (NP, HP, and IV). Serum phosphate, PTH and FGF23 on day 0 were comparable between Sham and Nx rats in the NP, HP and IV groups, but on day 7 PTH and FGF23 were significantly higher in Nx rats in all groups. This result might be due to the degree of kidney injury preventing detection of a significant difference between Sham and Nx rats on day 0 under each loading condition. Only IV loading resulted in a significantly higher serum phosphate level in Nx rats compared to Sham rats on day 7, suggesting that IV loading was the strongest phosphate loading condition in the study. The significant increase in FGF23 in Nx+NP rats compared with Sham+NP rats suggests that prolonged kidney injury might contribute to regulation of FGF23. A further study is needed to clarify the effect of kidney injury on FGF regulation.

While 1,25(OH)_2_D directly stimulates FGF23 expression in bone, FGF23 suppresses 1,25(OH)_2_D production mediating 1α-hydroxylase and 24-hydroxylase activities, suggesting a closed regulatory feedback loop [26]. Thus, administration of calcitriol to uremic rats dose-dependently increases FGF23 [22] and a positive correlation between calcitriol administration and FGF23 has been shown in dialysis patients [27]. We cannot rule out the possibility that 1,25(OH)_2_D mediates the response of IV phosphate loading. However, the serum 1,25(OH)_2_D level decreases as kidney dysfunction progresses, whereas FGF23 increases [3], [4], and the increase in FGF23 levels during CKD precedes the decrease in serum 1,25(OH)_2_D levels [5]. Moreover, phosphate induces catabolism of 1,25(OH)_2_D due to activation of 24-hydroxylase [28]. These observations suggest that phosphate loading is preferable for inactivation of 1,25(OH)_2_D. Therefore, serum 1,25(OH)_2_D is unlikely to have played an important role in FGF23 regulation in the present study.

PTH has been shown to increase FGF23 in animal studies [16], [17]. Dialysis patients with severe secondary hyperparathyroidism who underwent parathyroidectomy showed a decrease in FGF23 [29], and FGF23 levels correlated positively with PTH levels in these patients [30]. However, PTH has been proposed to be mainly regulated by Ca and 1,25(OH)_2_D, rather than FGF23, in a rat model of progressive renal failure [31]. In the present study, the change in PTH was imperceptible, probably due to conservation of serum Ca levels, and FGF23 was unlikely to have been regulated by the minimal change in PTH.

In kidney tissues, 1α-hydroxylase and NPT-2a expression was decreased and 24-hydroxylase expression was increased in both Nx+HP and Nx+IV rats, with similar changes in expression in the two groups. These results suggest that the FGF23 elevated by IV phosphate loading was bioactive, similarly to that after oral loading.

Berndt et al. demonstrated the presence of a rapid adaptation mechanism for phosphate loading from the intestine in normal kidney function, suggesting that intestinal factors for phosphate excretion might be present [32]. In the study, direct phosphate loading from the intestine increased FEP within 30 minutes, even though the levels of PTH and other phosphatonins such as FGF23 did not alter in this period. In our study, FEP in oral loading in Sham and Nx rats was significantly increased on day 1, in parallel with a significant increase in FGF23. The inconsistency of FGF23 variation compared with the previous report was probably due to the difference in the loading method or the observation period. Kidney function may also mask the regulation of FGF23 due to phosphate loading. Intravenous phosphate loading on day 1 showed a similar trend to oral loading, indicating another pathway specific to renal phosphate excretion that acts rapidly in response to intravenous loading.

We were unable to clarify the precise mechanisms through which IV phosphate loading increases FGF23. Since both oral and IV phosphate loading increased FGF23, it is likely that an increase in serum phosphate contributed to the increase in FGF23, but direct evidence for this effect is lacking [33]. Thus, serum phosphate effects on FGF23 might be mediated indirectly by bone mineralization, which could account for the delay in FGF23 elevation by serum phosphate. The continuous high serum phosphate levels produced by intravenous phosphate loading may be sensed in the bone, which leads to FGF23 production to counteract the phosphate load. This indicates the presence of a feedback system that may be different from the system based on oral phosphate loading. In kidney injury, in which phosphate is easily overloaded, the feedback system may become clearer, or perhaps the regulation of FGF23 may be beyond the serum phosphate levels. Further studies are warranted to determine the molecules that have a key role in the feedback system of phosphate metabolism.

In conclusion, this study is the first to show that continuous intravenous phosphate loading has a similar effect to that of oral phosphate loading in increasing bioactive FGF23. This phenomenon is clearer under conditions produced by a kidney injury in which phosphate is easily overloaded.
